# Bone Marrow-Liver-Spleen Type of Large B-Cell Lymphoma Associated with Hemophagocytic Syndrome: A Rare Aggressive Extranodal Lymphoma

**DOI:** 10.1155/2017/8496978

**Published:** 2017-08-01

**Authors:** Kirill A. Lyapichev, Jennifer R. Chapman, Oleksii Iakymenko, Offiong F. Ikpatt, Uygar Teomete, Sandra Patricia Sanchez, Francisco Vega

**Affiliations:** ^1^Division of Hematopathology, Department of Pathology and Laboratory Medicine, University of Miami and Sylvester Comprehensive Cancer Center, Miami, FL, USA; ^2^Department of Radiology, University of Miami and Sylvester Comprehensive Cancer Center, Miami, FL, USA; ^3^Division of Hematology-Oncology, Department of Medicine, University of Miami and Sylvester Comprehensive Cancer Center, Miami, FL, USA

## Abstract

Recently, an unusual subtype of large B-cell lymphoma (LBCL) with distinctive clinicopathologic features has been recognized; it is characterized by involvement of bone marrow with or without liver and/or spleen, but no lymph node or other extranodal sites, usually associated with fever, anemia, and hemophagocytic lymphohistiocytosis (HLH). Because of this distinctive clinical presentation, it has been designated “bone marrow-liver-spleen” (BLS) type of LBCL. To date there is only one series of 11 cases of BLS type of LBCL with detailed clinical, pathologic, and cytogenetic data. Herein, we describe a case of BLS type LBCL presenting with associated HLH in a 73-year-old female. The bone marrow core biopsy showed cytologically atypical large lymphoma cells present in a scattered interstitial distribution and hemophagocytosis and infrequent large lymphoma cells were seen in the bone marrow aspirate smears. Circulating lymphoma cells were not seen in the peripheral blood smears. The patient underwent treatment with chemotherapy (R-CHOP) but unfortunately passed away 2 months after initial presentation. BLS type of LBCL is a very rare and clinically aggressive lymphoma whose identification may be delayed by clinicians and hematopathologists due to its unusual clinical presentation and pathologic features.

## 1. Introduction

Diffuse large B-cell lymphoma (DLBCL) is the most common non-Hodgkin lymphoma (NHL) accounting for almost 40% of cases [1]. DLBCL is an aggressive tumor that usually presents with rapidly enlarging lymph nodes or extranodal masses. Secondary bone marrow (BM) involvement occurs in approximately 10%–16% of patients with DLBCL, NOS; however BM is an unusual site to establish the initial diagnosis of DLBCL [2, 3]. In addition to stage IV DLBCL, NOS, subtypes of DLBCL with relatively frequent BM involvement include intravascular large B-cell lymphoma (IVLBCL), splenic marginal zone lymphoma (splenic MZL), and T-cell/histiocyte-rich large B-cell lymphoma (THRLBCL) [1]. Recently a new subtype of DLBCL with frequent BM involvement and distinctive clinical and pathologic features has been recognized [4]. This lymphoma presents with BM involvement with or without involvement of the liver and/or spleen, often accompanied with severe anemia (100%), thrombocytopenia, hemophagocytic lymphohistiocytosis (HLH) (64%), and elevated lactate dehydrogenase (LDH) level in the absence of lymphadenopathy or other extranodal masses and with no prior history of lymphoma [4]. HLH is an uncommon a life-threatening disease of severe hyperinflammation caused by uncontrolled proliferation of activated lymphocytes and macrophages, characterized by proliferation of morphologically benign lymphocytes and macrophages that secrete high amounts of inflammatory cytokines. The current (2008) diagnostic criteria for HLH are a molecular diagnosis consistent with HLH (pathologic mutations of PRF1, UNC13D, or STX11) or fulfillment of five out of the eight criteria: (1) fever (>100.4°F, >38°C), (2) splenomegaly, (3) cytopenias (affecting at least two of three lineages in the peripheral blood: hemoglobin < 9 g/dL, platelets < 100 ×  10^9^/L, or Neutrophils < 1 ×  10^9^/L), (4) hypertriglyceridemia and/or hypofibrinogenemia (≤150 mg/100 ml), (5) ferritin ≥ 500 ng/ml, (6) hemophagocytosis in the bone marrow, spleen, or lymph nodes, (7) low or absent natural killer cell activity, and (8) soluble CD25 (soluble IL-2 receptor) >2400 U/ml (or per local reference laboratory) [5].

Because of its distinctive clinical presentation, this aggressive B-cell neoplasm has been named “bone marrow-liver-spleen” (BLS) type of LBCL (LBCL-BLS). Here, we present a case of LBCL-BLS associated with haemophagocytic syndrome in a 73-year-old female and compare it with previously published cases. Due to the limited availability of published accounts of this rare entity, we believe it is important to document our case in order to add to our understanding of this rare but clinically aggressive lymphoma.

## 2. Case Report

### 2.1. Clinical Presentation

A 73-year-old Hispanic female was admitted to our institution with weakness, fever, thrombocytopenia (70 ×  10^9^/L), neutropenia, elevated level of LDH (1010 U/L) and ferritin (>2000 ng/ml), and anemia (Hb 6.3 g/dL) of unclear etiology that required blood transfusions. She had no significant past medical history except for diabetes mellitus (DM).

**Figure 1:**
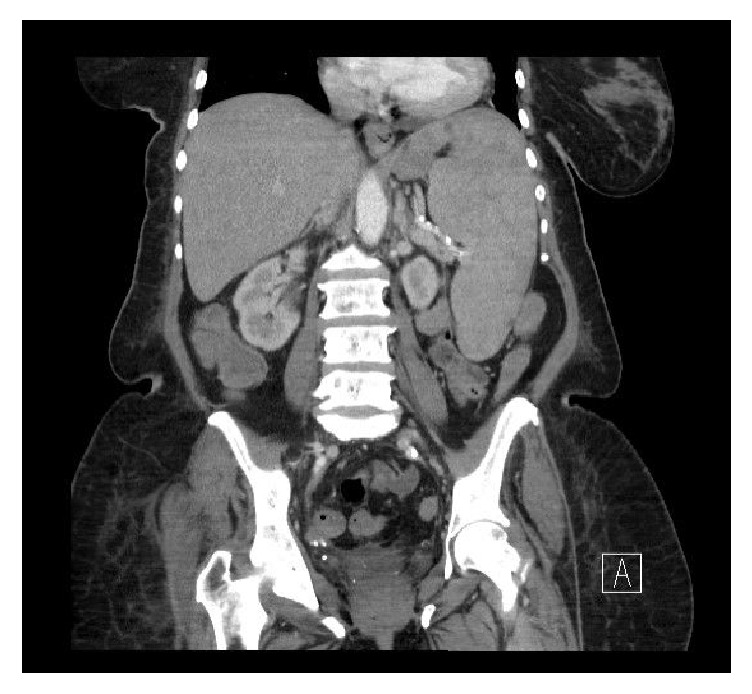
Abdominal and pelvic CT scan. Marked hepatomegaly and splenomegaly are identified. Spleen demonstrates numerous areas of heterogeneous hypoenhancement.

Computed tomography (CT) scan at the time of admission revealed no evidence of mediastinal, hilar, or other systemic lymphadenopathy but showed marked enlargement of liver and spleen (Figure 1). The spleen showed numerous heterogeneous areas of hypoenhancement, ranging between 2 and 5 cms, consistent with a neoplasm and suspicious for lymphoma. The adrenal glands were grossly within normal limits. Lung windows demonstrated patency of the trachea and main bronchi. No definite discrete pulmonary nodules or masses were identified. Osseous windows showed no definite lytic, sclerotic, or destructive lesions.

### 2.2. Pathologic Findings

**Figure 2:**
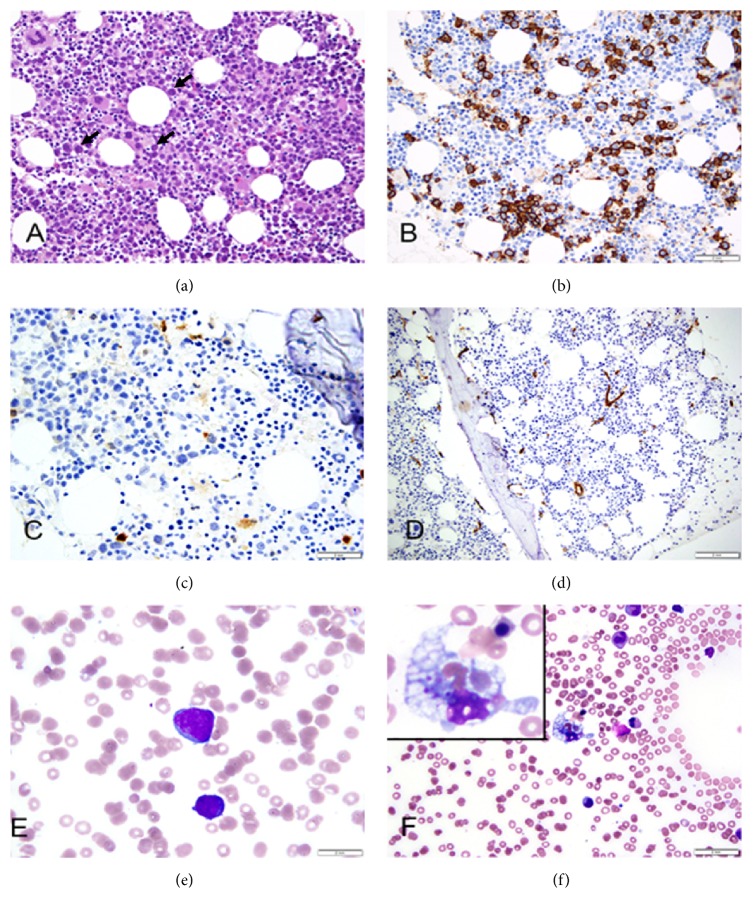
Histopathologic features. BM biopsy shows patchy interstitial infiltration of large lymphoma cells (arrows) without sinusoidal involvement (H&E; 400x) (a). By immunohistochemistry (IHC), the tumor cells are positive for CD20 (400x) (b) and negative for CD30 (400x) (c). CD34 highlights the capillary endothelial cells and confirms the lack of intravascular involvement by tumor cells (400x) (d). Lymphoma cells are large in size with large nuclei, high nuclear to cytoplasmic ratios, and open chromatin with one to several prominent nucleoli (Wright Geimsa; 100x) (e). BM aspirate smear shows histiocytic hemophagocytosis characterized by RBCs engulfing by histiocytes (Wright Geimsa; 100x) (f).

**Figure 3:**
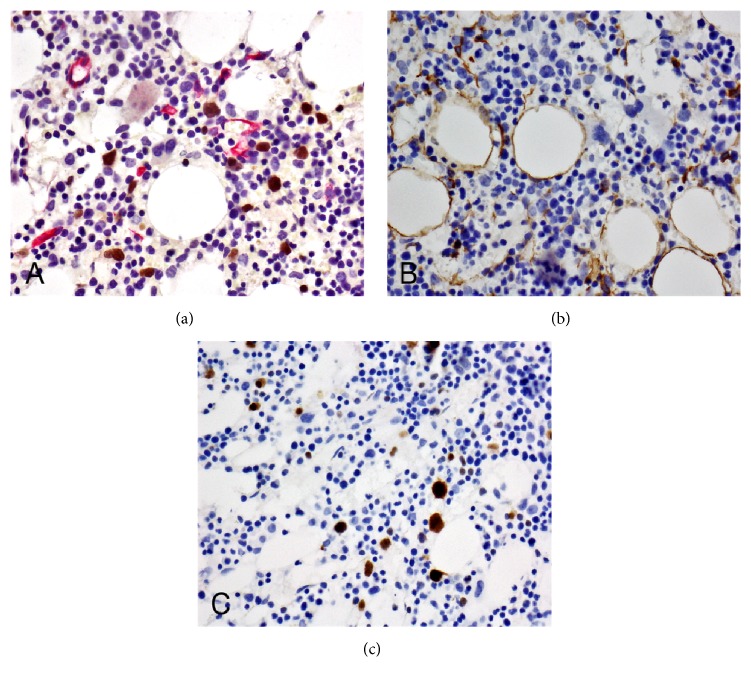
Histopathologic features. BM biopsy shows patchy interstitial infiltration of large, PAX5 positive lymphoma cells without sinusoidal (CD34 positive) involvement (400x) (a). By IHC, less than 30% of tumor cells are positive for CD10 (400x) (b) and more than 30% positive for MUM1 (400x) (c).

**Figure 4:**
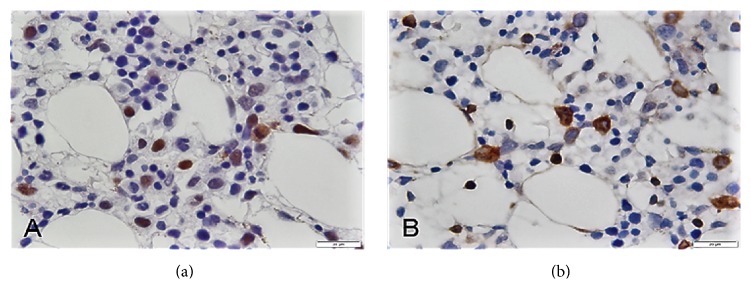
Histopathologic features. BM biopsy shows a subset of the tumor cells positive for MYC (100x) (a) and for BCL2 (100x) (b).

A BM biopsy was performed to investigate the etiology of the symptomatic anemia and thrombocytopenia. The BM core biopsy demonstrated a marrow cellularity of 60% with scattered large atypical lymphoid cells (Figure 2(a)). These cells were either scattered individually or formed loose clusters and represented approximately 10% of the total marrow cellularity (Figure 2(b)). Large clusters or sheets of lymphoma cells were not seen. Immunohistochemical studies showed that the tumor cells expressed pan-B-cell markers CD20 and CD79a and were negative for CD3, CD4, CD8, CD56, CD57, PD1, CD30, TdT, glycophorin, MPO, and CD117 (Figures 2(b) and 2(c)). CD20 and CD79a highlighted the interstitial distribution of the tumor cells. Tumor cells inside the marrow sinusoids were not seen (Figure 2(b)). Similarly, a CD34 immunostain and dual PAX5 with CD34 immunostains failed to show tumor cells inside the vessels. The lack of intrasinusoidal and intravascular tumor excluded the diagnosis of IVLBCL (Figures 2(d) and 3(a) and Table 2). As per the Hans algorithm based on the expression of CD10, BCL6, and MUM1, our case was classified into the nongerminal center B-cell-like (non-GCB) subgroup. IHC showed CD10 reactivity in less than 30% of tumor cells and MUM1 reactivity in more than 30% of tumor cells (Figures 3(b) and 3(c)). Additionally, BM biopsy was stained for MYC and for BCL2 and showed a subset of positive tumor cells (Figures 4(a) and 4(b)). The lymphoma cells were negative for Epstein–Barr virus-encoded RNAs (EBER). Small reactive T-cells were distributed throughout the marrow and consisted of a mixed population of CD4 and CD8 positive T-cells. Importantly, nodules rich in T-cells associated with the lymphoma cells were not seen and therefore the diagnosis of THRLBCL was also excluded (Table 2).

Individual large, atypical lymphoid cells were also recognized on the aspirate smears (Figure 2(e)). In addition, there were numerous histiocytes demonstrating hemophagocytosis throughout the aspirate smears (Figure 2(f)). Mild trilineage dyspoiesis was seen. Some of the dyspoietic granulocytes had toxic changes. A peripheral blood smear showed no circulating lymphoma cells.

Flow cytometry immunophenotypic analysis was performed on BM specimen and was negative for the presence of monoclonal B cells or increased blasts. Conventional karyotyping was negative for any clonal abnormalities and showed a diploid female karyotype, 46;XX [6]. Immunoglobulin heavy chain (IgH) and kappa light chain PCR assays performed in BM aspirate material confirmed the presence of a monoclonal B-cell population. This discrepancy between flow cytometry results and molecular analysis could be explained by poor representation of the tumor cells during flow cytometry analysis and high sensitivity of PCR assay. T-cell receptor (TCR) gamma PCR assay was negative for T-cell clonality.

Combining the pathologic findings with the clinical presentation, the patient was diagnosed with LBCL-BLS type. The neoplasm was associated with severe anemia, hepatosplenomegaly, and HLH syndrome. A biopsy of the spleen and the liver was not performed but based on the radiological studies both organs were considered involved by lymphoma.

### 2.3. Treatment and Clinical Course

After diagnosis, treatment with R-CHOP was immediately started. Approximately two months (51 days) after initiation of therapy the patient developed septic shock due to chemotherapy induced neutropenia and passed away.

## 3. Discussion

LBCL-BLS type is distinctive aggressive LBCL with characteristic clinicopathological features and aggressive clinical behavior that has been relatively recently recognized [4]. Patients usually present with fever, anemia of unclear etiology, thrombocytopenia, and increased LDH. On imaging studies splenomegaly or hepatosplenomegaly is typical and many small (usually less than 3 cm) hypodense nodules can be identified. Bone marrow biopsy usually reveals hemophagocytosis and an interstitial distribution of large lymphoma cells with a mature B-cell phenotype. Importantly, the lymphoma cells are not involving vascular lumina or sinusoids, differentiating this presentation from that of intravascular large B-cell lymphoma. To date, there are no specific genetic abnormalities and viral associations identified.

Nonnodal, primary LBCL involving BM with or without involvement of liver and/or spleen and no other extranodal involvement has been variably published in the literature [7–16]. However, given that this type of LBCL is not currently defined in the WHO classification, previous studies which included different types of lymphomas have been inconsistent in their reports of pathologic features and clinical outcomes. These previous reports seem to describe clinically similar lymphomas presenting with marrow involvement with separation into different categories based on additional site of involvement (BM only, spleen and BM or spleen, liver and BM).

To find previously reported cases of LBCL-BLS similar to ours, during the literature search we selected only studies of LBCL with initial manifestation in the BM, liver, and/or spleen (BLS type) that lacked any lymph node or other extranodal involvement and included genetic and survival information. Additionally, to make our search more specific we excluded diagnoses of IVLBCL, splenic marginal cell lymphoma with transformation, primary bone LBCL, and THRLBCL and used “Large B-cell lymphoma, bone marrow, liver, and spleen” as the keywords during literature search by PubMed. Using these criteria, we found only two publications that seemed similar to the case of LBCL-BLS that we describe [4, 14].

One of these, a study by Iioka et al. [14], describes 10 cases of DLBCL confined to BM, spleen, and liver, as evidenced by the uniformly increased uptake of fluorodeoxyglucose (FDG) on positron emission tomography combined with computed tomography (PET/CT). They retrospectively reviewed the clinical records of patients with aggressive B-cell lymphoma who were diagnosed and treated in their institution between 2011 and 2015. The inclusion criteria were as follows: patients whose clinical stage was determined on the basis of a FDG-PET/CT imaging study; patients whose disease showed the uniformly increased uptake of the tracer in the BM with or without diffuse uptake in the spleen and/or liver, but lacked uptake in the lymph nodes; and patients in whom a BM examination was performed for the diagnosis. Burkitt lymphoma, mantle cell lymphoma, the transformation of low-grade B-cell lymphomas, and any leukemia/lymphoma entity known to arise primarily in the BM were excluded. This study might represent 10 cases of LBCL-BLS but the authors do not specifically discriminate it from IVLBCL [14].

The BM biopsies were available in 9 out of 10 cases. Histology revealed lymphoma cells that were large with a moderate amount of cytoplasm, large vesicular nuclei, and one or more prominent nucleoli. Immunohistochemistry showed that lymphoma cells were positive for CD20 and CD79a. CD5 was positive in two cases showing the intrasinusoidal infiltration pattern; CD5 positivity was confirmed by flow cytometry. BCL2 was positive in eight cases. Per the Hans algorithm based upon the expression of CD10, BCL6, and MUM1, all cases were classified into the nongerminal center B-cell-like (non-GCB) subgroup. But they did not discriminate their cases from IVLBCL (did not perform CD34 IHC, to see intrasinusoidal involvement). Additionally, authors agreed that they will need to perform comparative studies incorporating histopathology and findings on FDG-PET/CT due to distinguish LBCL-BLS from IVLBCL. A different study by Yeh et al. reported detailed clinical, pathologic, and cytogenetic data in a series of LBCL-BLS whose clinical and histopathologic features were similar to those of our case [4]. In looking at the histopathologic features and clinical outcomes of the patients in the Yeh et al. series and our own, LBCL-BLS type seems to be a distinct clinicopathologic type of LBCL which has an aggressive clinical course and poor survival (Table 1). These lymphomas are characterized by variable age at presentation: 26–80 years, predominantly male population (M : F, 8 : 4), fever (100%), anemia (100%), thrombocytopenia (83%), increased LDH level (92%), bone marrow involvement (100%) with HLH syndrome (67%), nongerminal center phenotype (83%, as established by Hans algorithm), splenomegaly (100%), and hepatomegaly (25%) in the absence of lymphadenopathy and with no prior history of lymphoma. Additionally, it seems that the patient's survival rate depends at least in part on rapid lymphoma diagnosis and adequate and prompt treatment. Five patients (42%) in the previously reported series died in the first two and a half weeks after diagnosis (from 3 to 17 days); the patient we present died of lymphoma-related sepsis after only 51 days despite rapid lymphoma diagnosis and treatment initiation.

In Table 2 we compare LBCL-BLS as defined in this manuscript (LBCL involving BM, liver, and/or spleen but lacking intravascular or sinusoidal involvement and lacking nodal or other extranodal disease) with intravascular large B-cell lymphomas (Western and Asian types) and THRLBCL [1]. The lymphoma most similar to LBCL-BLS that is currently WHO-defined is IVLBCL, at least from the clinical perspective. Splenic MZL and THRLBCL might have similar clinical presentation too but usually they are very different pathohistologically and missing HLH. The lymphoma cells in splenic MZL are smaller and usually infiltrate BM with mixed pattern: combining nodular, paratrabecular, or diffuse with a distinctive intrasinusoidal component. Chromosomal translocation involving CDK6 gene at 7q21 as well as allelic loss of 7q31-32 is seen in a significant subset of the splenic MZL cases; NOTCH 2 and KLF2 mutations also might be present [17]. THRLBCL commonly involves liver, spleen, and bone marrow in addition to lymph node. In bone marrow, THRLBLC is characterized by multinodular aggregates rich in T-cells often associated with histiocytes and scattered large atypical lymphoid cells.

IVLBCL is a rare type of extranodal LBCL characterized by the selective growth of lymphoma cells within the lumina of capillaries and small/medium sized vessels. There are two IVLBCL subtypes: Western type characterized by symptoms related to the main organ involvement, predominantly central nervous system (CNS) or skin, and an Asian type in which the patients present with multiorgan failure, hepatosplenomegaly, pancytopenia, fever, B symptoms, and HLH. The second type (Asian type) of IVLBCL is closer in clinical presentation (pancytopenia, HLH, and skin and central nervous system involvements are uncommon) to LBCL-BLS with the pathologic difference being that lymphoma cells have a striking intravascular and intrasinusoidal distribution in Asian type IVLBCL. B symptoms are very common (55–76%) and lymphoma cells are more often of activated B-cell type in both types of IVLBCL as well as in LBCL-BLS [1]. Polymerase chain reaction (PCR) studies in both entities show negative results for Epstein–Barr virus (EBV) and human herpesviruses 6 and 8 (HHV6 and HHV8) infections in vast majority of cases. It is unclear at this time whether IVLBCL, particularly Asian type, and LBCL-BLS type, as reported herein, are variants of the same high-grade form of LBCL. A high incidence of HLH is also an important and characteristic feature of both IVLBCL and LBCL-BLS type. Although the precise mechanism underlying this lymphoma-associated HLH syndrome remains unclear, overproduction of TNF-*α*, INF-*γ*, IL-1a, IL-6, IL-10, and other cytokines by nonfunctional cytotoxic T/NK-cells as well as macrophage hyperactivation is thought to play a crucial role [18–20].

In addition to the difference in lymphoma cell distribution (intravascular versus BM interstitial), the Asian type of IVLBCL and LBCL-BLS type have also not yet shown overlapping molecular genetic features, supporting that these two lymphoma types are different. Specifically, Asian type IVLBCL has been associated with abnormalities of 19q13 and 8p21, which have not been found in LBCL-BLS type thus far [4, 6, 21]. LBCL-BLS type, alternatively, has shown multiple chromosomal defects including t(8;14)(q24;q32), dup(14)(q24;q32), inv(14)(q32), t(3;6), trisomy 18, add(19)(p13), del(8)(p22), del(3)(q21), and add(7)(p22) [4, 22, 23]. However, given the limited number of available cases in the literature at this time, additional studies are needed with specific comparisons of these two lymphoma types to determine their relatedness, or lack thereof. Finally, lack of CD29 (*β*-1 integrin) and CD54 (ICAM-1) in IVLBCL may be potentially useful diagnostic feature. Defects (downregulation) in these protein receptors on the lymphoma cells might prevent the neoplasm from invasion through vascular wall and contribute to the intravascular location.

## 4. Conclusion

We describe an unusual and clinically aggressive extranodal large B-cell lymphoma involving the BM, spleen, and liver (LBCL-BSL). This lymphoma appears to be a distinct but rare and controversial entity that may be a specific type of extranodal DLBCL, NOS [1]. However, aside from lymphoma cell distribution within vascular spaces and marrow sinusoids, this lymphoma has clinical features similar to those of Asian type IVLBCL. Importantly, the clinical presentation and disease course are particularly severe with rapid disease progression and high mortality rate during first weeks to months after initial symptoms. Overlapping clinical and morphological features can make it challenging to differentiate LBCL-BLS from more common lymphomas including splenic marginal zone lymphoma with large cell or prolymphocytic transformation, THRLBCL and, particularly, IVLBCL (Asian type). Gene expression profiling studies comparing these entities may help in developing an understanding of the biology of these lymphomas and their relatedness as well as in predicting effective treatment protocols.

## Figures and Tables

**Table 1:** Clinical features of patients with large B cell lymphoma initially manifesting in the bone marrow. Patients N1–N9 are from National Cheng Kung University Hospital; patients M1 and M2 are from University of Texas M.D. Anderson Cancer Center; and patient UM1 is from University of Miami. H/S: hepatomegaly/splenomegaly; HS: haemophagocytic syndrome; IPI: international prognostic index; C/T: chemotherapy; CHOP: cyclophosphamide, doxorubicin, vincristine, and prednisolone (E, epirubicin); R-ESHAP: rituximab, etoposide, methylprednisolone, cytarabine, and cisplatin; CVAD: Cyclophosphamide, vincristine, doxorubicin hydrochloride (Adriamycin), and dexamethasone; PBSCT: peripheral blood stem cell transplantation; F: female; M: male [4].

Case	Age/sex	Fever	H/S	Cytopenia	LDH	HS	Radiological splenic mass (>3 cm)	Treatment	IPI	Outcome (days)
N1	26/F	+	−/+	Leukopenia and anemia	293	−	—	C/T (CHOP*∗*3, R-ESHAP), PBSCT	2	Alive (1560)
N2	73/F	+	−/+	Anaemia and thrombocytopenia	293	+	Many small hypodense nodules	C/T (CHOP*∗*1, R-CHOP*∗*5)	3	Dead (784)
N3	44/M	+	−/+	Anaemia and thrombocytopenia	745	−	Many small hypodense nodules	C/T (CEOP*∗*1, R-CEOP*∗*5, R-ESHAP*∗*4), PBSCT	2	Dead (551)
N4	54/M	+	−/+	Pancytopenia	727	+	—	C/T (CHOP*∗*6)	2	Dead (285)
N5	80/M	+	−/+	Anaemia and thrombocytopenia	319	+	Wedge-shaped hypodense infarct	—	4	Dead (17)
N6	72/F	+	−/+	Anaemia and thrombocytopenia	1255	−	—	—	4	Dead (8)
N7	76/M	+	−/+	Anaemia and thrombocytopenia	426	+	—	—	4	Dead (6)
N8	61/M	+	−/+	Anaemia and thrombocytopenia	160	+	—	—	3	Dead (4)
N9	75/M	+	−/+	Anaemia and thrombocytopenia	4464	+	Many small hypodense nodules	—	4	Dead (4)
M1	69/M	+	−/+	Anemia	482	−	—	C/T (R-CVAD*∗*6)	4	Dead (84)
M2	60/M	+	+/+	Anaemia and thrombocytopenia	2266	+	Many small hypodense nodules	C/T (R-CVAD*∗*6)	4	Dead (224)
UM1	73/F	+	+/+	Thrombocytopenia, neutropenia and anemia	1010	+	Numerous heterogeneous areas (2 to 5 cm)	C/T (R-CHOP with steroids)	4	Dead (51)

**Table 2:** Differential diagnosis of LBCL-BLS and subtypes of IVLBCL and TCHRLBCL.

	LBCL-BLS	IVLBCL (Western type)	IVLBCL (Asian type)	TCHRLBCL
CD20	+	+	+	+
CD79a	+	+	+	+
CD3	−	−	−	Background T cells
CD5	−	38%+	38% +	Background T cells
EBV	−	−	−	+
HLH	+	+/−	+	−
Peripheral lymph node involvement	−	−	−	+
Involvement of vascular lumina by lymphoma cells	−	+	+	−
Specific Alterations	None	None	19q13 and 8p21	−
Organ of involvement	BM, liver and/or spleen	CNS or skin	BM	Liver, spleen, BM
T cell rich lymphoid nodules	−	−	−	+
HCV infection association	−	−	−	−
Bone marrow, rich in T cell nodules	−	−	−	+
